# Association between clinician team structure and continuous pulse oximetry overuse among infants hospitalized with bronchiolitis: An Eliminating Monitor Overuse Trial substudy

**DOI:** 10.1002/jhm.70420

**Published:** 2026-08-09

**Authors:** Andrew S. Kern‐Goldberger, Jennifer A. Faerber, Rui Xiao, Spandana Makeneni, Kimberly Albanowski, Canita R. Brent, Halley Ruppel, Tina V. Halley, Kaitlyn M. McQuistion, Enrique F. Schisterman, Rinad S. Beidas, Christopher P. Bonafide, For the Pediatric Research in Inpatient Settings (PRIS) Network

**Affiliations:** ^1^ Division of Pediatric Hospital Medicine Cleveland Clinic Children's Hospital Cleveland Ohio USA; ^2^ Cleveland Clinic Lerner College of Medicine at Case Western Reserve University Cleveland Ohio USA; ^3^ Data Science and Biostatistics Unit, Children's Hospital of Philadelphia Research Institute Philadelphia Pennsylvania USA; ^4^ Department of Biostatistics, Epidemiology, and Informatics, Perelman School of Medicine University of Pennsylvania Philadelphia Pennsylvania USA; ^5^ Section of Hospital Medicine, Children's Hospital of Philadelphia Philadelphia Pennsylvania USA; ^6^ Clinical Futures, Children's Hospital of Philadelphia Research Institute Philadelphia Pennsylvania USA; ^7^ Department of Family and Community Health University of Pennsylvania School of Nursing Philadelphia Pennsylvania USA; ^8^ Division of Hospital Medicine, Children's National Hospital Washington District Columbia USA; ^9^ Division of Hospital Medicine, Medical College of Wisconsin Milwaukee Wisconsin USA; ^10^ Department of Medical Social Sciences, Feinberg School of Medicine Northwestern University Chicago Illinois USA; ^11^ Department of Pediatrics, Perelman School of Medicine University of Pennsylvania Philadelphia Pennsylvania USA; ^12^ Penn Implementation Science Center (PISCE) University of Pennsylvania Philadelphia Pennsylvania USA

## Abstract

Continuous pulse oximetry (cSpO_2_) monitoring for hospitalized children with bronchiolitis not requiring supplemental oxygen is an established form of medical overuse. We examined whether clinician team structure influences cSpO_2_ overuse. In this secondary analysis of the Eliminating Monitor Overuse (EMO) Trial (NCT05132322), we compared overuse by frontline clinician type (resident physician vs. attending physician or advanced practice provider [APP]) during the active cSpO_2_ deimplementation and sustainment phases using multivariable mixed‐effects logistic regression. Among 4167 inpatient observations in 36 hospitals, 61% had their frontline care provided by residents, 27% by attendings, and 10% by APPs. In adjusted analyses, the odds of overuse were 47% higher among patients with frontline care delivered by attendings compared with residents (adjusted odds ratio: 1.47 [95% confidence interval [CI]: 1.12–1.93], *p* = .01), with no difference observed for APPs compared with residents. These findings suggest that cSpO_2_ overuse may be attenuated when residents deliver frontline patient care rather than attendings alone.

## INTRODUCTION

Continuous pulse oximetry (cSpO_2_) use in children with bronchiolitis who are not receiving supplemental oxygen is discouraged by national guidelines and widely recognized as medical overuse,^1^ contributing to increased alarm burden and healthcare utilization without improving patient outcomes.^2^ Prior studies have elucidated factors associated with cSpO_2_ overuse, including age and prematurity, overnight monitoring,^3^ and low bronchiolitis volume hospital units.^4^ However, it is unknown whether the clinician team structure—specifically, the type of frontline clinician (i.e., responsible for placing orders and serving as the first contact for nurses)—influences cSpO_2_ overuse. Our objective was to compare cSpO_2_ overuse by team structure (subintern, intern, or resident physicians [“residents”]; advanced practice providers [APPs]; or attending physicians in frontline clinician role). We hypothesized that teams with residents serving as the frontline clinician would have higher rates of cSpO_2_ overuse than teams with frontline care delivered by attendings or APPs, potentially reflecting greater safety concerns among less experienced trainees.

## METHODS

### Study design and setting

We performed a substudy of the Eliminating Monitor Overuse (EMO) SpO_2_ Trial, a cluster‐randomized hybrid type 3 effectiveness‐deimplementation trial conducted in the Pediatric Research in Inpatient Settings (PRIS) Network. This trial compared the effects of an unlearning‐only strategy (educational outreach and audit and feedback) versus an unlearning plus substitution strategy (electronic health record clinical decision support) on sustainment of cSpO2 deimplementation in children with bronchiolitis in room air; the study protocol and statistical analysis plan were previously published.^5^, ^6^ The three phases of the trial were baseline measurement (Phase 1, P1), active deimplementation (P2), and sustainment (P3, after withdrawal of the unlearning‐based strategies). Data for this substudy were collected during P2 (January 20, 2023–May 20, 2023) and P3 (December 1, 2023–March 31, 2024).

### Patient population

The parent trial included infants with a primary diagnosis of bronchiolitis aged 8 weeks through 23 months who are nonintensive care, nonemergency department, nonstep‐down inpatient units cared for by a generalized inpatient service. Infants with major comorbidities, coronavirus disease 2019 (COVID‐19), and gestational age < 28 weeks were excluded.^5^

### Data collection

In the EMO SpO_2_ Trial, continuous pulse oximetry monitoring status was ascertained through cross‐sectional observational data collection rounds conducted by staff at each hospital. In addition to chart review, this included visualizing the cSpO_2_ waveform at the bedside or using secure remote viewing platforms. Specific to this substudy, during the P2 and P3 phases, we incorporated into the REDCap data collection form a prompt asking staff to complete the following optional question: “Which type of provider is the front‐line clinician caring for this patient (i.e., responsible for placing orders, first contact for nurses)?” with response options of subintern, intern or resident physician (“residents”), fellow, APPs (e.g., nurse practitioner or physician assistant), attending physicians, or other.

### Data analysis

We summarized study variables overall and stratified by clinician team structure by count and percentage. Study variables included (a) patient or observation‐level covariates used in prior analyses in the EMO SpO_2_ portfolio (age combined with preterm birth, time since weaning from supplemental oxygen or flow, documented history of apnea or cyanosis during the present illness, prior intensive care unit stay during present hospitalization, presence of an enteral feeding tube, history of neurologic impairment, and nighttime observation)^3^, ^4^; (b) study phase (P2 or P3); (c) intervention arm (education + audit‐and‐feedback vs. education + audit‐and‐feedback + clinical decision support [CDS]); and (d) additional prespecified hospital‐level covariates: presence of pre‐existing CDS for cSpO_2_ use before the start of the trial, and hospital type (freestanding or not freestanding). We also examined clinician team structure at the hospital level by describing the percentage of observations corresponding to each team structure within each hospital.

In the primary analysis comparing cSpO_2_ overuse by clinician team structure, we fit univariable (unadjusted) and multivariable (adjusted) hierarchical mixed‐effects logistic regression models, with hospital‐ and patient‐specific random intercepts to account for clustering of observations within patients and hospitals. The covariates adjusted in the multivariable modeling included patient‐ and hospital‐level variables. Cross‐tabulation was used to evaluate the extent of overlap and sparse cell counts among the binary hospital‐level variables (intervention arm, hospital type, presence of pre‐existing CDS) as an indicator of potential collinearity (Supporting Information S1: Appendix Table 1). To evaluate robustness, we conducted sensitivity analyses adjusting for (a) intervention arm alone, (b) intervention arm and hospital type, and (c) intervention arm and pre‐existing CDS, and (d) all three hospital‐level variables. We used predictive margins to estimate the adjusted overuse rates for each clinician team structure. All analyses were performed using Stata version 19.

## RESULTS

We identified 4167 observations of infants with bronchiolitis across 36 hospitals. Clinician team structure was recorded for 4134 observations (99.2%). Of these, 2550 (61%) involved frontline care provided by residents, 1145 (27%) by attendings, 427 (10%) by APPs, 9 (0.2%) by fellows, and 3 (0.1%) by other clinician types (Table 1). After excluding 83 observations with missing covariates, 4051 observations were included in the adjusted regression analysis. The distribution of clinician team structure by hospital is shown in the Supporting Information S2: Appendix Figure.

**Table 1:** Characteristics of study population and continuous pulse oximetry overuse.

	Patient observations, *n* (column %)^a^,^b^			
	Intern/resident (*n* = 2550)	Attending physician (*n* = 1145)	Advanced practice provider (*n* = 427)	Total (*N* = 4134)^a^
Study phase				
Phase 2 (active deimplementation)	1299 (50.9)	544 (47.5)	180 (42.2)	2034 (49.2)
Phase 3 (Sustainment)^c^	1251 (49.1)	601 (52.5)	247 (57.8)	2100 (50.8)
Combined age and preterm status				
8 weeks–5 months and not preterm	653 (25.6)	330 (28.9)	109 (25.5)	1097 (26.5)
8 weeks–5 months and preterm	53 (2.0)	27 (2.4)	9 (2.1)	89 (2.2)
6–11 months and not preterm	903 (35.4)	412 (36.0)	160 (37.5)	1477 (35.7)
6–11 months and preterm	76 (3.0)	29 (2.5)	15 (3.5)	120 (2.9)
12–17 months and not preterm	481 (18.9)	204 (17.8)	76 (17.8)	764 (18.5)
12–17 months and preterm	32 (1.3)	17 (1.5)	2 (0.5)	51 (1.2)
18–24 months and not preterm	310 (12.2)	116 (10.1)	55 (12.9)	483 (11.7)
18–24 months and preterm	27 (1.1)	7 (0.6)	1 (0.2)	35 (0.8)
Time off supplemental oxygen or flow				
Never on O_2_	471 (18.5)	262 (22.9)	127 (29.7)	862 (20.9)
Off 1‐ < 2 h	221 (8.7)	52 (4.5)	25 (5.9)	300 (7.3)
Off 2‐ < 4 h	376 (14.7)	156 (13.6)	48 (11.2)	580 (14.0)
Off 4‐ < 6 h	230 (9.0)	122 (10.7)	38 (8.9)	395 (9.6)
Off 6‐ < 12 h	374 (14.7)	175 (15.3)	71 (16.6)	621 (15.0)
Off 12‐ < 24 h	495 (19.4)	225 (19.7)	73 (17.1)	794 (19.2)
Off >=24 h	377 (14.8)	153 (13.4)	45 (10.5)	576 (13.9)
Neurological impairment				
No	2474 (97.0)	1,115 (97.4)	421 (98.6)	4022 (97.3)
Yes	37 (1.5)	12 (1.0)	4 (0.9)	53 (1.3)
Prior ICU stay during present hospitalization				
No	2020 (79.2)	972 (84.9)	375 (87.8)	3378 (81.7)
Yes	492 (19.3)	155 (13.5)	50 (11.7)	698 (16.9)
Presence of enteral feeding tube				
No	2389 (93.7)	1,093 (95.5)	399 (93.4)	3893 (94.2)
Yes	161 (6.3)	52 (4.5)	28 (6.6)	241 (5.8)
Overnight observation				
No	2097 (82.2)	893 (78.0)	347 (81.3)	3344 (80.9)
Yes	453 (17.8)	252 (22.0)	80 (18.7)	790 (19.1)
Hospital type				
Not freestanding (*N* = 10 hospitals)	429 (16.8)	247 (21.6)	56 (13.1)	735 (17.8)
Freestanding (*N* = 26 hospitals)	2121 (83.2)	898 (78.4)	371 (86.9)	3399 (82.2)
Presence of pre‐existing CDS				
No (*N *= 9 hospitals)	533 (20.9)	68 (5.9)	9 (2.1)	611 (14.8)
Yes (*N* = 27 hospitals)	2017 (79.1)	1,077 (94.1)	418 (97.9)	3,523 (85.2)
Intervention arm				
Education + A&F (*N* = 18 hospitals)	1489 (58.4)	450 (39.3)	319 (74.7)	2264 (54.8)
Education + A&F + CDS (*N* = 18 hospitals)	1061 (41.6)	695 (60.7)	108 (25.3)	1870 (45.2)
Continuously monitored with pulse oximetry, observed				
No	1780 (69.8)	814 (71.1)	331 (77.5)	2930 (70.9)
Yes	770 (30.2)	331 (28.9)	96 (22.5)	1204 (29.1)

Abbreviations: A&F, audit‐and‐feedback; CDS, clinical decision support; ICU, intensive care unit; O_2_, oxygen.

^a^ A total of 4167 observations were recorded in the study. Clinician team structure was documented in 4134 observations. Observations in which the frontline clinician was classified as fellow (*n* = 9), other (*n* = 3), or missing (*n* = 33) are omitted from the table due to low sample sizes.

^b^ Column percentages may not sum to 100% due to missing data.

^c^ Following withdrawal of unlearning‐based implementation strategies (audit‐and‐feedback and educational outreach).

cSpO_2_ overuse occurred in 1204 observations (29.1%). In unadjusted mixed‐effects logistic analyses, odds of overuse were 31% higher among patients with frontline care led by attendings compared with residents (odds ratio [OR] = 1.31 [95% confidence interval [CI]: 1.03–1.67], *p* = .03). In multivariable mixed‐effects modeling after adjustment for covariates, odds of overuse were 47% higher among patients with frontline care led by attendings compared with residents (adjusted odds ratio [aOR] 1.47: [95% confidence interval [CI]: 1.12–1.93], *p* = .01). This corresponds to a 5.2‐percentage point difference (95% CI: 1.6–8.9) in adjusted overuse rates (Table 2). Results were nearly identical across models adjusting for distinct combinations of hospital‐level covariates (Supporting Information S1: Appendix Table 2). There was no difference in cSpO_2_ overuse among patients with frontline care led by APPs compared with residents (aOR: 0.91, 95% CI: 0.61–1.35, *p* = .65).

**Table 2:** Continuous pulse oximetry monitoring by clinician team structure.

Frontline clinician role	Number of observations (*N* = 4051)^a^	Unadjusted overuse, % of observations (95% CI)^b^	Adjusted overuse, % of observations (95% CI)^c^
Intern/resident	2490	32.1 (26.7–37.4)	30.7 (26.0–35.5)
Attending	1124	36.5 (30.3–42.7)	36.0 (30.4–41.5)
Advanced practice provider	425	30.9 (23.7–38.1)	29.5 (23.2–35.9)

Abbreviation: CI, confidence interval.

^a^ Number of observations in primary analysis after excluding 83 observations with missing covariates. Results corresponding to fellow (*n* = 9) or other (*n* = 3) clinician type in frontline role are omitted from the table due to low sample size.

^b^ Mixed‐effects model using random intercepts to account for hospital‐ and patient‐level clustering, without adjustment for covariates.

^c^ Mixed‐effects model using random intercepts to account for hospital‐ and patient‐level clustering, adjusted for patient‐level covariates (age combined with preterm birth, time since weaning from supplemental oxygen or flow, documented history of apnea or cyanosis during the present illness, prior intensive care unit stay during present hospitalization, presence of an enteral feeding tube, history of neurologic impairment, and nighttime observation), study phase, intervention arm, and presence of pre‐existing clinical decision support (CDS). Results corresponding to alternative models adjusting for hospital type, and both hospital type and clinical decision support, are presented in Supporting Information S1: Appendix Table 2.

## DISCUSSION

In this 36‐hospital study of over 4000 cSpO2 observations, cSpO_2_ monitoring overuse was more likely among patients with frontline care delivered by attendings compared with residents. Contrary to our hypothesis, this finding—robust to confounding by other factors—suggests that trainee involvement in frontline patient care may attenuate cSpO2 overuse. Based on prior work characterizing alarm rates associated with guideline‐discordant versus guideline‐concordant cSpO_2_ monitoring (6.7 vs. 0.5 alarms/hour),^7^ the observed 5.2‐percentage point reduction in cSpO_2_ overuse among resident‐led teams translates to 7.7 fewer alarms per patient‐day, with the potential to mitigate costs and downstream cascade effects of overuse such as reducing alarm fatigue and length of stay.^8^

Prior qualitative studies of clinicians have suggested that trainees may be less comfortable deimplementing cSpO_2_ due to limited clinical experience.^9^, ^10^ Our data challenge this assumption and suggest alternative explanations. First, resident physicians’ relative inexperience and exposure to diverse care settings during their training may facilitate deimplementation. Because training environments emphasize inquiry and frequent feedback, residents often have less entrenched practices and greater openness to change,^11^ potentially increasing receptiveness to evidence‐based guidelines and new workflows. Second, relative to attendings, residents may have less hierarchical interactions with nurses, which could facilitate nurse‐driven discontinuation of cSpO_2_ monitoring.^10^ This dynamic may help explain why resident‐led teams had lower cSpO_2_ overuse compared with attending‐led teams but not APP‐led teams, as APPs may similarly have less hierarchical interactions with nurses. APPs have also demonstrated more favorable attitudes toward reducing practice variation and standardizing care,^12^ suggesting a professional culture consistent with the principles emphasized in contemporary residency training.^13^ Third, prior work specific to cSpO_2_ use in bronchiolitis^10^ and in other clinical contexts^14^ has shown that resident engagement in evidence‐based practices is highly motivated by the behavior of more senior physicians. This may make residents more responsive to the education and audit‐and‐feedback strategies embedded in the trial. At the same time, residents’ position within the clinical hierarchy^15^ relative to the attendings serving as trial site leads may make them less likely to act upon any psychological reactance (resistance to threatened autonomy)^16^ experienced in response to deimplementation messaging. Future work should formally evaluate these mechanisms and tailor implementation strategies to clinician team structures.

More broadly, this study contributes to a growing literature on the role of distinct inpatient practice models in ensuring quality and mitigating overuse. Multiple systematic reviews and observational studies have demonstrated that teaching services staffed by residents produce equivalent, and at times higher quality, inpatient care than attending‐only teams.^17^, ^18^, ^19^, ^20^ To our knowledge, however, no prior study has specifically examined the association between clinician team structure and care quality in the context of a quality improvement or implementation study. Despite concerns that residents are difficult to engage in quality improvement or have negative attitudes toward such work,^21^ our findings support the potential value of residents as the foundation of a learning health system.^22^

Our study has several limitations. First, information on clinician team structure was not recorded during Phase 1 of the trial, limiting our ability to assess baseline differences in the absence of active deimplementation efforts or evaluate longitudinal sustainment of deimplementation.^5^ Second, clinician team structure was uniform at some hospitals while mixed at others (Supporting Information S2: Appendix Figure). Although we accounted for hospital‐level clustering and adjusted for hospital‐level variables, unmeasured hospital‐ or unit‐level factors may still influence the association between team structure and cSpO_2_ overuse. Third, we did not evaluate characteristics of the attending physicians. It is possible that attendings supervising resident teams are different from attendings working in frontline roles with respect to their clinical experience, comfort with reducing cSpO_2_ monitoring, and length of employment at the hospital, which may influence their alignment with existing institutional practices and culture. Finally, similar to other projects in the EMO portfolio, data collection relied on convenience sampling of cSpO_2_ use, which may introduce selection bias.

## CONCLUSIONS

Inpatient care delivered by teams with resident physicians in a frontline role demonstrated lower cSpO_2_ overuse than frontline attending teams, a finding that persisted after adjustment for other factors. Future research should investigate the mechanisms by which residents attenuate cSpO_2_ overuse through mixed‐methods inquiry, evaluate whether resident‐led teams achieve greater sustainment of overuse reduction, and determine how to tailor implementation strategies to clinician roles. Finally, examining whether these findings extend to other forms of medical overuse will inform broader deimplementation efforts.

## CONFLICT OF INTEREST STATEMENT

Dr. Beidas receives funding from the National Institutes of Health, Northwestern Medicine, National Psoriasis Foundation, American Foundation for Suicide Prevention, West Health Institute, Sanofi, and Pfizer for her research. Dr. Beidas is principal at Implementation Science & Practice, LLC and serves as co‐Editor‐In‐Chief for Implementation Science. She is currently an appointed member of the National Advisory Mental Health Council and serves on the scientific advisory board for AIM Youth Mental Health Foundation, Klingenstein Third Generation Foundation, and the American Foundation for Suicide Prevention. She was previously an appointed member of the NASEM study, “Blueprint for a national prevention infrastructure for behavioral health disorders,” served on the scientific and advisory board for Optum Behavioral Health, received consulting fees from United Behavioral Health and OptumLabs, and received royalties from Oxford University Press. All activities are outside of the submitted work. Dr. Enrique Schisterman has received consulting fees from Merck for work on causal inference methods. He is Editor‐in‐Chief of the *American Journal of Epidemiology*. None of these activities are directly related to the work presented here.

## Supporting information

EMO Substudy Appendix Tables 1 and 2_R1.

Supplementary Information

## Data Availability

The data that support the findings of this study are available from the corresponding author upon reasonable request.
