# Supplementary material for: Association between clinician team structure and continuous pulse oximetry overuse among infants hospitalized with bronchiolitis: An Eliminating Monitor Overuse Trial substudy
Source: J Hosp Med. 2026 Aug 9;21(11):1279–85. doi: 10.1002/jhm.70420 (PMC13458530; doi:10.1002/jhm.70420)
Supplement: Supplementary file 1 [file JHM-21-1279-s002.docx]

**APPENDIX**

**Appendix Table 1. Relationship Between Hospital-Level Covariates**

Appendix Table 1a. Relationship Between Hospital Type and Pre-Existing Clinical Decision Support

| **Hospital Type** | **Pre-Existing Clinical Decision Support** | | |
| --- | --- | --- | --- |
|  | No | Yes | Total |
| Not Freestanding |  |  |  |
| Observations, n (column %) | 214 (34.8) | 527 (14.8) | 741 (17.8) |
| Hospitals, N | 4 | 6 | 10 |
| Freestanding |  |  |  |
| Observations, n (column %) | 401 (65.2) | 3,025 (85.2) | 3,426 (82.2) |
| Hospitals, N | 5 | 21 | 26 |
| Total |  |  |  |
| Observations, n | 615 | 3,552 | 4,167 |
| Hospitals, N | 9 | 27 | 36 |

*Chi-square *p*<0.001 comparing observations by Pre-Existing Clinical Decision Support and Hospital Type

Appendix Table 1b. Relationship Between Hospital Type and Intervention Arm

| **Hospital Type** | **Intervention Arm** | | |
| --- | --- | --- | --- |
|  | Education + A&F | Education + A&F + CDS | Total |
| Not Freestanding |  |  |  |
| Observations, n (column %) | 263 (11.5) | 478 (25.3) | 741 (17.8) |
| Hospitals, N | 4 | 6 | 10 |
| Freestanding |  |  |  |
| Observations, n (column %) | 2,017 (88.5) | 1,409 (74.7) | 3,426 (82.2) |
| Hospitals, N | 14 | 12 | 26 |
| Total |  |  |  |
| Observations, n | 2,280 | 1,887 | 4,167 |
| Hospitals, N | 18 | 18 | 36 |

*Chi-square *p*<0.001 comparing observations by Intervention Arm and Hospital Type

** Abbreviations: A&F, audit-and-feedback; CDS, clinical decision support

Appendix Table 1c. Relationship Between Pre-Existing Clinical Decision Support and Intervention Arm

| **Pre-Existing Clinical Decision Support** | **Intervention Arm** | | |
| --- | --- | --- | --- |
|  | Education + A&F | Education + A&F + CDS | Total |
| No |  |  |  |
| Observations, n (column %) | 257 (11.3) | 358 (19.0) | 615 (14.8) |
| Hospitals, N | 4 | 5 | 9 |
| Yes |  |  |  |
| Observations, n (column %) | 2,023 (88.7) | 1,529 (81.0) | 3,552 (85.2) |
| Hospitals, N | 14 | 13 | 27 |
| Total |  |  |  |
| Observations, n | 2,280 | 1,887 | 4,167 |
| Hospitals, N | 18 | 18 | 36 |

*Chi-square *p*<0.001 comparing observations by Intervention Arm and Pre-Existing Clinical Decision Support

** Abbreviations: A&F, audit-and-feedback; CDS, clinical decision support

**Appendix Table 2. Effect of Clinician Team Structure on Continuous Pulse Oximetry Overuse by Model^a,b^**

| Frontline Clinician Role  [ref = Intern/Resident] | Statistical Measure  (rows) | Model 1 | Model 2 | Model 3 ^c^ | Model 4 | Model 5 |
| --- | --- | --- | --- | --- | --- | --- |
| Fellow | OR | 7.759* | 8.808* | 8.887* | 8.825* | 8.879* |
|  | 95% CI | 1.342, 44.865 | 1.257, 61.743 | 1.269, 62.211 | 1.258, 61.889 | 1.269, 62.148 |
|  | P-value | 0.02 | 0.03 | 0.03 | 0.03 | 0.03 |
|  | | | | | | |
| Advanced Practice Provider | OR | 0.928 | 0.900 | 0.912 | 0.901 | 0.912 |
|  | 95% CI | 0.645, 1.335 | 0.606, 1.338 | 0.614, 1.355 | 0.606, 1.339 | 0.614, 1.355 |
|  | P-value | 0.69 | 0.60 | 0.65 | 0.61 | 0.65 |
|  | | | | | | |
| Attending  Physician | OR | 1.311** | 1.457** | 1.472** | 1.459** | 1.472** |
|  | 95% CI | 1.029,  1.672 | 1.111, 1.910 | 1.123, 1.930 | 1.112, 1.913 | 1.123, 1.930 |
|  | P-value | 0.03 | 0.01 | 0.01 | 0.01 | 0.01 |
|  | | | | | | |
| Other | OR | 1.403 | 2.533 | 2.572 | 2.528 | 2.575 |
|  | 95% CI | 0.073, 26.941 | 0.122, 52.693 | 0.124, 53.521 | 0.121, 52.620 | 0.124, 53.580 |
|  | P-value | 0.82 | 0.55 | 0.54 | 0.55 | 0.54 |

** p<.01, * p<.05

^a^ Results have been exponentiated

^b^ All models use random intercepts to account for hospital- and patient-level clustering

^c^ Model reflected in results section of manuscript body

Abbreviations: ref= reference; OR = odds ratio; CI = confidence interval; CDS = clinical decision support

Model 1: Unadjusted
Model 2: Adjusted for Phase, Intervention Arm, and Patient/Observation-Level Covariates
Model 3: Adjusted for Phase, Intervention Arm, Patient/Observation-Level Covariates, and CDS Presence
Model 4: Adjusted for Phase, Intervention Arm, Patient/Observation-Level Covariates, and Hospital Type

Model 5: Adjusted for Phase, Intervention Arm, Patient/Observation-Level Covariate, CDS Presence, and Hospital Type
